# Exploring the feasibility of exercise training during (neo)adjuvant chemotherapy in women with early-stage breast cancer: a pilot study examining immune and metabolic outcomes

**DOI:** 10.1007/s00520-026-11179-8

**Published:** 2026-09-24

**Authors:** Cristina Crespo-García, Francesco Bettariga, Dennis R. Taaffe, John P. Campbell, Carolyn J. Peddle-McIntyre, Emily Jeffery, Andrew D. Redfern, Catherine A. Rinaldi, Daniel A. Galvao, Robert U. Newton

**Affiliations:** 1 Exercise Medicine Research Institute, Edith Cowan University, Perth, WA 6027 Australia; 2 School of Medical and Health Sciences, Edith Cowan University, Perth, WA Australia; 3 School of Human Performance, Rehabilitation and Population Health, INSIGHT Research Institute, University of Technology Sydney, Sydney, NSW, Australia; 4 Department for Health, University of Bath, Bath, UK; 5 Curtin School of Population Health, Curtin University, Perth, WA Australia; 6 School of Medicine and Pharmacology, University of Western Australia, Perth, WA Australia; 7 Centre for Microscopy, Characterisation & Analysis, University of Western Australia, Perth, WA Australia; 8 School of Human Movement and Nutrition Sciences, University of Queensland, Brisbane, QLD Australia

**Keywords:** Breast cancer, Chemotherapy, Exercise oncology, CD8 + T cells, Feasibility

## Abstract

**Purpose:**

To examine the feasibility and exploratory immunological, metabolic, and patient-reported effects of a supervised exercise intervention during (neo)adjuvant chemotherapy in women with early-stage breast cancer.

**Methods:**

This non-randomised pilot study examined a 12-week supervised combined aerobic and resistance exercise programme during (neo)adjuvant treatment. Participants self-selected into the exercise intervention, and as no eligible participants opted for the control group, a convenience control group was subsequently recruited for blood biomarker comparisons. Sessions were performed 2–3 times per week—with exercise individually tailored according to treatment-related side effects and by progressively adjusting intensity to maintain a target rating of perceived exertion (RPE) of 12–16 (Borg 6–20 scale). Eleven patients (49.1 ± 14.4 years) completed the intervention and six (57.5 ± 5.4 years) comprised the convenience control group. Outcomes included feasibility (retention, attendance and adherence rates), immune cells, cytokines, metabolic biomarkers, body composition (lean and fat mass), health-related quality of life, and fatigue.

**Results:**

Retention was 11/13 participants (84.6%), while attendance and adherence were 83.6 ± 23.1% and 77.9 ± 24.9%, respectively. Significant differences in immune cell subsets were observed, particularly within the cluster of differentiation 8 (CD8) + T cell compartment. Compared with controls, exercisers showed a greater increase in CD8 + effector memory re-expressing CD45RA (EMRA) cells (*p* = 0.019, Hedges’ *g* = 1.26) and a reduction in CD8 + naïve T cells (*p* = 0.029, Hedges’ *g* = − 1.16). Exercise prevented the insulin rise observed in controls (*p* = 0.034). Compared to baseline, body weight and fat mass were maintained, with a signal toward increased lean mass (0.8 kg, *p* = 0.093). Finally, exercise improved global quality of life (+ 16.7 points, *p* = 0.031) and showed a signal toward reduced fatigue (FACIT-F TOI + 8.4 points, *p* = 0.063).

**Conclusions:**

The supervised exercise programme was feasible among participants who enrolled in the intervention. Exploratory analyses suggest exercise may promote cytotoxic immune cell differentiation and mitigate metabolic dysregulation while significantly improving global quality of life. Larger trials are needed to confirm these findings.

**Supplementary Information:**

The online version contains supplementary material available at https://doi.org/10.1007/s00520-026-11179-8.

## Introduction

Breast cancer remains one of the most prevalent malignancies worldwide and a leading cause of cancer-related mortality [1]. Advances in treatment, including chemotherapy, have significantly improved survival rates [2]. However, given that immune function plays a critical role in chemotherapy response [3], interventions that support immune health, such as exercise, may enhance treatment outcomes.

Preclinical evidence shows that aerobic exercise reduces tumour incidence, growth, and metastasis through both intrinsic tumour effects (e.g. reduced proliferation and angiogenesis) and systemic changes, including improved metabolism, vascularisation, and immune modulation [4], with the latter being a key mechanism [5, 6]. Exercise induces a rapid mobilisation of immune cells into the circulation via shear stress and β2-adrenergic receptor activation [4], facilitating the redistribution of natural killer (NK) cells and cluster of differentiation 8 (CD8) + T cells into peripheral tissues, enhancing immunosurveillance [6, 7]. Moreover, regular physical activity and exercise in healthy populations promote a more competent immune system by reducing dysfunctional T cell populations, including senescent and exhausted T cells [6, 8], and increasing naïve T cells [9], which are essential for recognising tumour neoantigens [5].

Exercise-induced myokines further contribute to these adaptations: IL-7 and IL-15 sustain naïve and memory T cells and promote NK cell differentiation [6, 10], whereas IL-6 has been shown to facilitate NK cell infiltration into tumours in mice models, thereby enhancing local antitumour immunity [7, 11]. In animal models, these systemic effects translate into reduced tumour growth through increased immune infiltration of CD8 + T and NK cells [11, 12] and alleviation of immunosuppressive conditions in the tumour microenvironment (TME), fostering an environment more permissive to antitumour immunity [5, 6, 13].

Epidemiological studies link higher physical activity with lower all-cause and breast cancer–specific mortality [14, 15]. Recent evidence suggests that these associations are primarily supported by studies of aerobic exercise, whereas evidence for muscle-strengthening exercise remains limited and heterogeneous, although available studies suggest potential benefits for survival outcomes [16, 17]. Moreover, randomised controlled trials (RCTs) have demonstrated that exercise interventions during chemotherapy improve quality of life, fatigue, fitness, and body composition in patients with breast cancer [18–21]. However, the specific immunomodulatory effects of exercise in patients with breast cancer during chemotherapy remain insufficiently characterised. A recent meta-analysis in breast cancer patients and survivors found no significant effects of exercise on broad circulating tumour-relevant immune subsets (CD4 +, CD8 +, and NK cells), likely due to limited phenotyping and protocol heterogeneity [22]. Only one recent trial by Ubink et al. [23] assessed the effects of an exercise programme on a more detailed panel of immune populations, underscoring the need for further research on specific T cell and NK cell subsets during chemotherapy.

To address this research gap, the fasting and exercise (FASTEX) pilot study was designed to investigate the independent effects of a supervised exercise programme and a fasting-mimicking diet (FMD) as potential adjunct strategies during chemotherapy in patients with early-stage breast cancer. The trial included two independent intervention groups, each compared with a shared usual-care control group. Here, we report the results of a supervised exercise programme, while results from the FMD group will be reported separately. The primary outcome was feasibility, while secondary outcomes focused on the effects on peripheral immune cell phenotypes, cytokines, metabolic biomarkers, body composition, and patient-reported outcomes.

## Methods

### Study design

The FASTEX study was a non-randomised, controlled preliminary efficacy trial. The study was designed to examine the independent effects of each intervention compared with a shared usual-care control group, and no direct comparison between the two intervention arms was planned. Patients were informed about the study interventions and self-selected into either the supervised exercise or FMD intervention according to their preference. As no eligible participants opted for the usual-care control group, a convenience sample of control participants was subsequently recruited. To facilitate recruitment and minimise participant burden, control participants consented to provide blood samples only rather than undergoing the full study assessment. The study was approved by the South Metropolitan Health Service Human Research Ethics Committee (RGS0000006143) in Perth, Western Australia, and registered with the ANZCTR (ID 12623000996662; registration date 13/09/2023). Written informed consent was obtained from all patients. All procedures performed were in accordance with the South Metropolitan Health Service and Edith Cowan University guidelines and regulations.

### Patients

Eligible participants were ≥ 18 years old with histologically confirmed breast cancer undergoing (neo)adjuvant chemotherapy (± immunotherapy) and able to attend assessments and provide blood samples. Exclusion criteria included uncontrolled comorbidities, pregnancy, breastfeeding, ≥ 300 min/week habitual exercise, or medical contraindications to exercise. The upper limit of 300 min/week of exercise was selected to maximise recruitment rather than to restrict participation to insufficiently active individuals. In addition, baseline physical activity was assessed using the Godin Leisure-Time Exercise Questionnaire. The Godin Leisure Score Index is calculated from the self-reported weekly frequency of strenuous, moderate, and light leisure-time physical activity. Higher scores indicate greater levels of leisure-time physical activity. Participants were recruited from December 2023 to February 2025, from Sir Charles Gairdner Hospital, Fiona Stanley Hospital and Joondalup Health Campus, which serve the Perth metropolitan area. Exercise group participants underwent baseline and post-intervention testing at Edith Cowan University, whereas controls provided blood samples only.

### Intervention: exercise programme

Participants completed a 12-week supervised combined aerobic and resistance training programme (~ 60 min per session) at Vario Clinic, Edith Cowan University or Fiona Stanley Hospital. The programme was designed to include two supervised sessions during chemotherapy weeks and three sessions during non-chemotherapy weeks. As chemotherapy schedules varied between participants, the prescribed number of sessions was individualised accordingly, while attendance remained flexible according to participants’ symptoms and treatment tolerance. Exercise sessions were offered throughout the day, from morning to late afternoon. Session times were arranged flexibly in consultation with participants, taking into account their treatment schedules, work and personal commitments, and the availability of other participants attending supervised sessions. Participants did not receive any financial incentives for participation However, parking costs associated with attending supervised exercise sessions were reimbursed. Sessions included a 5–10 min warm-up (walking or stationary cycling), 15–20 min of aerobic exercise, 20–25 min of resistance training, and a 5-min cool-down (walking or stationary cycling). Intensity was progressively adjusted throughout the intervention to maintain participants within the prescribed target rating of perceived exertion (RPE) of 12–16 on the Borg 6–20 scale [24].

Resistance training followed a standard progression (2–4 sets, 8–10 reps) and targeted the major upper- and lower-body muscle groups using a combination of machine-based and free-weight. Exercises, including leg press, leg curl, leg extension, calf raises, chest press, seated row, lat pulldown, shoulder press, and upper-limb exercises such as biceps curls and triceps extensions. Load and total training volume were recorded to ensure progressive overload. Aerobic exercise was performed on a treadmill or stationary bike and individualised by adjusting treadmill speed and incline or cycling resistance according to each participant’s tolerance and perceived exertion to maintain the prescribed RPE of 12–16.

### Outcome measures

#### Feasibility

Feasibility was assessed through retention (participants completing the study/participants recruited), attendance (sessions attended/prescribed) and adherence (sessions completed as prescribed). Attendance and adherence were calculated among participants who completed the intervention, and the trial was considered feasible if retention reached 82% and attendance and adherence reached 75% [25]. Reasons for missed or modified sessions and for discontinuation of the intervention were recorded.

Serious adverse events derived from the exercise intervention (defined as life-threatening events or those requiring hospital admission or resulting in death), as well as non-serious adverse events, were documented by a study investigator and by patients using a logbook.

#### Biomarker analyses

##### Blood sample collection and storage

In the exercise group, blood samples were collected in a fasting state. Baseline blood samples were obtained 3–8 days after the first or second chemotherapy dose. Post-intervention samples were taken 3–8 days after a scheduled chemotherapy dose and 48–72 h after the final exercise training session to minimise the influence of acute exercise response. The interval between baseline and post-intervention sampling was 13–14 weeks. In the control group, due to logistical constraints related to chemotherapy scheduling, blood samples were not collected in a fasting state. Baseline samples were collected after 2–4 chemotherapy doses, typically 3 weeks post-infusion. Post-intervention samples were also typically collected 3 weeks after the last chemotherapy dose, with intervals between baseline and post-intervention sampling ranging from 3 to 12 weeks.

At each time point, 36 mL of blood was collected for peripheral blood mononuclear cell (PBMC) isolation and 10 mL for plasma and serum. Serum was allowed to clot for 30 min, and plasma and serum were centrifuged at 1600 × g for 10 min at 21°C, with supernatants stored at − 80°C. PBMCs were isolated by density gradient centrifugation using Leucosep tubes with Lymphoprep, followed by washing and red blood cell lysis. Cells were cryopreserved in FBS with 10% DMSO at − 1°C/min and stored at − 80°C before transferring to liquid nitrogen.

Baseline and end-of-intervention samples from each participant were analysed under the same experimental conditions and, where possible, within the same assay batch to minimise technical variability and reduce the risk of measurement bias.

##### Flow cytometry

PBMC surface markers were analysed using a 5-laser Cytek Aurora spectral flow cytometer. Approximately 1.5–2 × 10^6^ cells per panel were stained with FVS700 viability dye, blocked with 2% mouse serum, and incubated with fluorochrome-conjugated monoclonal antibodies.

Two antibody panels were used. Panel 1 was designed for T cell phenotyping, including differentiation, activation and senescence markers, as well as NK cell characterisation, and included CD3 BV605 (SK7), CD8 APC-H7 (HIT8A), CD4 BV786 (SK3), CCR7 BV711 (2-L1-A), CD45RA BB515 (HI100), CD95 BV480 (DX2), CD57 BV421 (NK-1), HLA-DR BUV395 (G46-6), CD56 RY586 (B159), and CD16 PE-Cy7 (3G8). Panel 2 was used to assess T cell exhaustion markers and included CD3 BV605 (SK7), CD8 APC-H7 (HIT8A), CD4 BV786 (SK3), TIM-3 Alexa 647 (7D3), and CTLA-4 PE (BNI3).

All antibodies were purchased from BD Biosciences (Oxford, UK). After staining, cells were fixed and stored overnight. Spectral unmixing was performed using unstained and single-stained reference controls with BD CompBeads, and fluorescence-minus-one (FMO) controls prepared from pooled PBMCs. Between 50,000 and 100,000 events were acquired per sample

##### Serum and plasma biomarkers

Glucose levels were measured in whole blood with a glucometer.

Insulin, IGF-1, IGFBP-1, IL-6, IL-7, and IL-15 were assessed in serum/plasma using enzyme-linked immunosorbent assays (ELISAs) according to manufacturer instructions (Human ELISA Kit, Abcam, Cambridge, UK).

#### Body composition

Whole-body lean mass (LM) and fat mass (FM) (kg), appendicular lean mass (ALM) (kg), lean mass percentage (LM%), body fat percentage (FM%), and visceral adipose tissue mass (VAT) (g) were measured at baseline and post-intervention by dual-energy X-ray absorptiometry (DXA) (Horizon A, Hologic, WA, USA).

#### Patient-reported outcomes

Self-administered questionnaires were used to assess fatigue (Functional Assessment of Chronic Illness Therapy (FACIT)-F) [26], cancer-related quality of life (European Organization for Research and Treatment of Cancer, Quality of Life Questionnaire, EORTC QLQ-C30) [27], breast cancer–specific quality of life (EORTC QLQ-BR23) [28], nutritional status (Patient-Generated Subjective Global Assessment Short Form, PG-SGA SF) [29], and physical activity (Leisure Score Index of the Godin Leisure-Time Physical Activity Questionnaire) [30]. Acceptability of the intervention was assessed using a 7-point Likert scale (1, not at all; 7, very much) and open-ended questions [31]. For each item, the frequency of responses per score was recorded, and the median score was calculated. Acceptability questions are detailed in Supplementary Table2.

#### Pathological response to neoadjuvant chemotherapy

The pathologic complete response (pCR) was assessed after the completion of neoadjuvant treatment, following surgical resection, according to the American Joint Committee on Cancer (AJCC) system [32].

### Statistical analysis

Given that this was a pilot study examining feasibility and preliminary efficacy, no formal sample size calculation was made. A target sample size of 13 participants per group was established based on recruitment feasibility within the available study timeframe and resources. Data were analysed using SPSS (v29.0, IBM, Armonk, NY) and R (v4.4.2, The R Foundation, Vienna, Austria). Normality was assessed with the Shapiro–Wilk test. For outcomes available only in the exercise group (e.g. body composition, fatigue, quality of life), within-group pre-post differences were tested using the paired *t*-test (or the Wilcoxon signed-rank test when non-normal distribution). For blood and immune markers, because sampling intervals differed between the exercise and control groups, we computed the time-normalised change Δweek = (post − pre)/weeks to account for differing intervals between groups. Between-group differences in Δweek were assessed using Welch’s *t*-test when normally distributed, or the Mann–Whitney *U* test otherwise. Given that this pilot trial was underpowered by design, both statistically significant differences (*p* < 0.05) and signals toward statistical significance (*p* < 0.10) are reported. Within-group analyses were restricted to markers showing between-group differences or signals and were considered exploratory post-hoc analyses to aid interpretation of the direction of change. Within each group, Δweek was compared with zero using a two-sided one-sample *t*-test or a one-sample Wilcoxon test when distributions were non-normal. For comparisons analysed using Welch’s and one-sample *t* tests, effect sizes were calculated as Hedges’ *g* with a small-sample correction and interpreted as small (|*g*|≥ 0.2), moderate (|*g*|≥ 0.5), or large (|*g*|≥ 0.8).

## Results

### Feasibility and acceptability

**Fig. 1:**
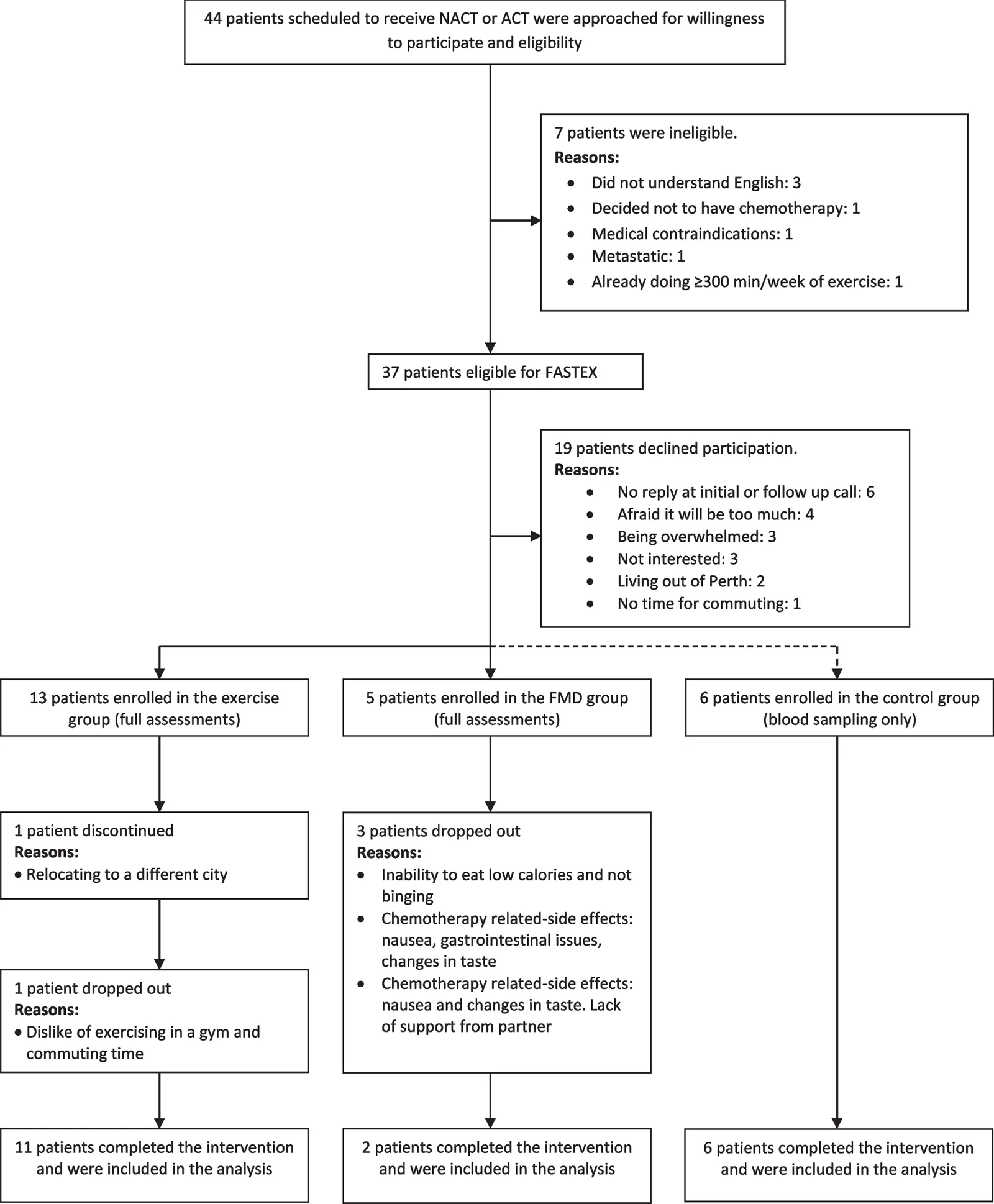
Consort diagram showing flow of patients

The flow of patients throughout the study is presented in Fig. 1. Of the 44 patients screened, 37 met eligibility criteria and were invited to participate. Thirteen enrolled in the exercise group, resulting in an uptake rate of 35.1%. Participants were recruited from three hospitals across the Perth metropolitan area, with exercise sessions delivered at two sites to improve accessibility. Two patients withdrew from the exercise intervention due to travel-related reasons. One participant relocated during the study, while the other reported that travelling to the exercise facility, together with a preference for exercising outdoors, influenced her decision to withdraw. The remaining eleven patients (84.6% retention rate) completed the 12-week programme and were included in the analyses. In addition, five patients chose to participate in the FMD arm, reported elsewhere. Six participants were subsequently recruited into the convenience control group and consented to provide blood samples only. Baseline demographic and clinical characteristics of patients in the exercise and control groups are presented in Table 1.

**Table 1:** Participant characteristics

Variable	Exercise group (*n* = 11) Mean ± SD or *N* (%)	Control group (*n* = 6) Mean ± SD or *N* (%)
Age (years)	49.1 ± 14.4	57.5 ± 5.4
Weight (kg)	70.0 ± 14.1	72.3 ± 19.6
Height (cm)	163.3 ± 6.3	160.0 ± 6.1
BMI (kg/m^2^)	26.4 ± 5.8	28.0 ± 6.0
Premenopausal	8 (72.7%)	1 (16.7)
Ethnicity		
White	8 (72.7%)	4 (66.7%)
Asian/pacific islander	3 (27.3%)	2 (33.3%)
Tumour grade		
Grade 2	3 (27.3%)	1 (16.7%)
Grade 3	8 (72.7%)	5 (83.3%)
Tumour stage		
Stage IA	3 (27.3%)	2 (33.3%)
Stage IB	0 (0%)	1 (16.7%)
Stage IIA	2 (18.2%)	1 (16.7%)
Stage IIB	4 (36.4%)	1 (16.7%)
Unknown	2 (18.2%)	1 (16.7%)
Receptor status		
ER/PR + HER2 −	7 (63.6%)	1 (16.7%)
ER/PR ± HER2 +	2 (18.2%)	4 (66.7%)
Triple-negative	2 (18.2%)	1 (16.7%)
Chemotherapy timing		
NACT	4 (36.4%)	3 (50.0%)
ACT	7 (63.6%)	3 (50.0%)
(Immuno)chemotherapy regimen		
ddAC-T-Carboplatin	1 (9.1%)	
AC-T-Carboplatin + pembrolizumab	1 (9.1%)	1 (16.7%)
AC-T	3 (27.3%)	
AC-TH		1 (16.7%)
TC	4 (36.4%)	1 (16.7%)
TCH	2 (18.2%)	3 (50.0%)
No. of chemotherapy doses		
≤ 6 (12–18 weeks	7 (63.6%)	4 (66.7%)
> 6 (20–24 weeks)	4 (36.4%)	2 (33.3%)
Godin Leisure Score Index		**-**
Active (≥ 24)	5 (45.5%)	
Moderately active (14–23)	3 (27.3%)	
Insufficiently active (< 14)	2 (18.2%)	
Nutritional status (PG-SGA SHORT FORM)		**-**
Low risk (0–1 points)	1 (9.1%)	
Moderate risk (2–3 points)	0 (0.0%)	
High risk (4–8 points)	7 (63.6%)	
Severe risk (≥ 9 points)	3 (27.3%)	**-**
Completed university	7 (63.6%)	
Employment status	4 (36.4%)	**-**
Full-time employed	2 (18.2%)	
Part-time employed	2 (18.2%)	
On leave from work	3 (27.3%)	
Unemployed/retired	4 (36.4%)	
Married	7 (63.6%)	**-**
Smoker		**-**
Current	0 (0.0%)	
Past	5 (45.5%)	
Current drinker	5 (45.5%)	**-**
Comorbidities		**-**
Hypertension	2 (18.2%)	
Arthritis	2 (18.2%)	
Diabetes	1 (9.1%)	
High cholesterol	1 (9.1%)	

*BMI* body mass index, *NACT* neoadjuvant treatment, *ACT* adjuvant treatment, *ER* oestrogen receptor, *PR* progesterone receptor, AC-T: doxorubicin (Adriamycin®), cyclophosphamide (Cytoxan®), paclitaxel (Taxol®); ddAC-T: dose dense doxorubicin (Adriamycin®), cyclophosphamide (Cytoxan®), paclitaxel (Taxol®); AC-TH: doxorubicin (Adriamycin®), cyclophosphamide (Cytoxan®), paclitaxel (Taxol®), and trastuzumab (Herceptin®); TC: docetaxel (Taxotere®) and cyclophosphamide (Cytoxan®); TCH: docetaxel (Taxotere®), carboplatin (Paraplatin®), and trastuzumab (Herceptin®); PG-SGA: patient-generated subjective global assessment. –, no data available. Current drinker, participant self-reported consuming alcohol at baseline (Yes to the question “Do you consume alcohol?”). Comorbidities were self-reported at baseline; no clinical assessments or diagnostic thresholds were applied

Mean attendance was 83.6 ± 23.1%. Most missed sessions were due to treatment-related side effects (69%; e.g. fatigue, gastrointestinal symptoms, pain), including one participant who attended only 8 of 32 sessions because of persistent physical and emotional ill health. Personal reasons accounted for 25.7% of missed sessions, medical appointments for 4.3%, and facility issues for 1.4%.

Mean adherence to the prescribed exercise was 77.9 ± 24.9%. Session modifications were required due to medical considerations (40%; e.g. PICC line, clipped node, pre-session blood tests), comorbidities (38%; e.g. plantar fasciitis, hypotension, post-surgical cording), or chemotherapy-related symptoms (22%). Two participants completed all sessions without modification.

Participants demonstrated consistent progression in resistance training load and volume, confirming effective implementation of progressive overload. On average, resistance training load increased by 10–60%, and total volume approximately doubled over 12 weeks (Supplementary Table 1). Leisure-time physical activity (Godin Leisure Score Index, arbitrary units) showed a non-significant increase from baseline to post-intervention (21.8 ± 12.4 to 26.6 ± 12.2; *p* = 0.115).

One transient episode of mild hypotension occurred during an exercise session in a participant with pre-existing hypertension who had taken antihypertensive medication; no other exercise-related adverse events were observed. Acceptability of the supervised exercise programme was high (Supplementary Table 2), with low median burden ratings (1/7) and highly positive evaluations for perceived reward, personal usefulness, and willingness to recommend the programme. Fatigue and treatment-related symptoms were the most commonly reported barriers, while other factors were rated low, indicating good overall feasibility. Open-ended responses highlighted logistical challenges (e.g. transport, parking), alongside perceived improvements in well-being, treatment tolerance, and the supportive exercise environment.

### Immune biomarkers

**Fig. 2:**
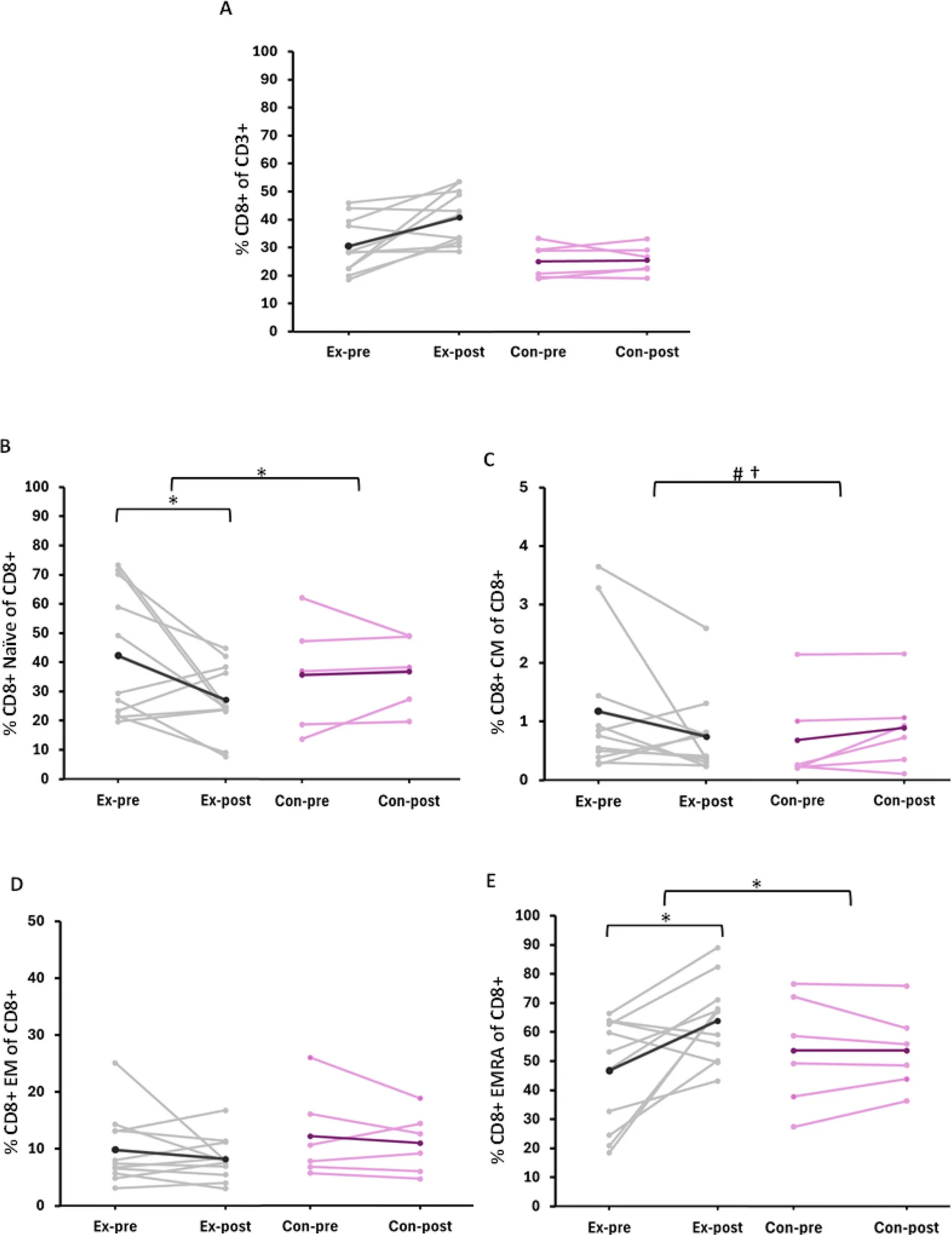

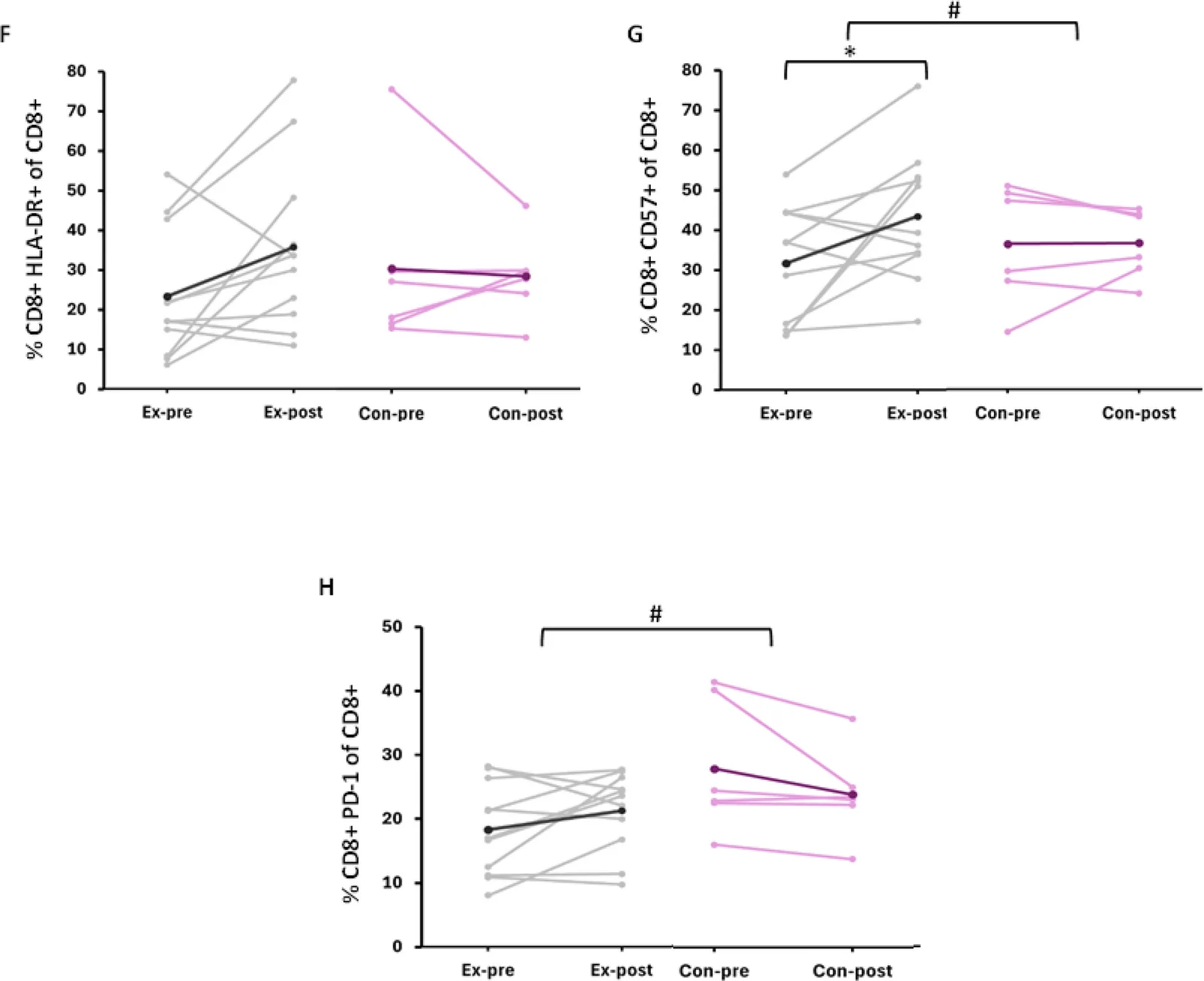
Changes in CD8 T cell subsets from pre- to post-intervention in the exercise and control groups. Changes in peripheral CD8 T cell subsets from baseline (pre) to end of intervention (post) in the exercise (grey, *n* = 11) and control (pink, *n* = 6) groups. Data are shown as individual values and group means. Ex: exercise; Con: control. **A** % CD8 + of CD3 + cells; **B** % CD8 + naïve; **C** % CD8 + CM; **D** % CD8 + EM; **E** % CD8 + EMRA; **F** % CD8 + HLA-DR +; **G** % CD8 + CD57 +; **H** % CD8 + PD-1 +. Signals toward significance (*p* < 0.10) are indicated by #, and statistically significant differences (*p* < 0.05) by *. *p*-values correspond to between-group comparisons and within-group comparisons of change scores normalised by the number of weeks between pre- and post-assessments. †*p*-values calculated using Mann–Whitney *U* test. Abbreviations: CM, central memory; EM, effector memory; EMRA, effector memory re-expressing CD45RA

Peripheral immune cell frequencies are shown in Fig. 2, Supplementary Figs. 2 and 3, and Supplementary Table 3.

Between-group analyses revealed significant differences within the CD8 + T-cell compartment. Specifically, the proportion of CD8 + EMRA cells increased in the exercise group compared with controls (*p* = 0.019, Hedges’ *g* = 1.26, large effect; Fig. 2E), while CD8 + naïve T cells were significantly lower (*p* = 0.029, Hedges’ *g* = − 1.16, large effect; Fig. 2B). Signals toward group differences were also observed for CD8 + CM (*p* = 0.050; Fig. 2C) and CD8 + naïve CD95 + T cells (*p* = 0.087, not shown), which decreased in exercisers but increased in controls. CD8 + CD57 + and CD8 + PD-1 + T cells tended to increase in the exercise group, while remaining stable or declining in controls (*p* = 0.053, Hedges’ *g* = 1.02, large effect and *p* = 0.050, Hedges’ *g* = 1.06, large effect, respectively; Fig. 2 G and H). Within the CD4 + compartment, a signal toward a group difference was observed for total CD4 + T cells, which decreased in exercisers and increased in controls (*p* = 0.097; Supplementary Fig. 2 A). In addition, controls showed a signal toward a greater reduction in CD4 + CTLA-4 + TIM-3 + cells compared to exercisers (*p* = 0.052, Hedges’ *g* = 1.05, large effect; Supplementary Table 3). Among NK-cells, there was a signal toward a greater increase in CD56brightCD16 + NK cells in controls compared to the exercise group (*p* = 0.079, Hedges’ *g* = − 0.92, large effect; Supplementary Fig. 3 C), while total NK cells, NKT-like cells and the rest of the subsets showed no significant differences (Supplementary Fig. 3).

Within-group analyses demonstrated more pronounced immunological changes in the exercise group, particularly within CD8 + T cells. Significant increases were observed in the proportion of CD8 + EMRA (*p* = 0.017, Hedges’ *g* = 0.80, large effect; Fig. 2E) and CD8 + CD57 + T cells (*p* = 0.044, Hedges’ *g* = 0.64, moderate effect; Fig. 2G), alongside reductions in the proportion of total CD4 + T cells (*p* = 0.008, Hedges’ *g* = − 0.92, large effect; Supplementary Fig. 2 A) and CD8 + naïve T cells (*p* = 0.040, Hedges’ *g* = − 0.66, moderate effect; Fig. 2B). Finally, no significant changes were observed in NK cell subsets (Supplementary Fig. 3).

In contrast, the control group exhibited limited within-group changes, with a significant reduction in CD4 + CTLA-4 + TIM-3 + cells (*p* = 0.019, Hedges’ *g* = − 1.17, large effect; Supplementary Table 3) and an increase in CD56brightCD16 + NK cells (*p* = 0.041, Hedges’ *g* = 0.94, large effect; Supplementary Fig. 3 C), and no other significant differences across immune parameters (Fig. 2, Supplementary Figs. 2–3 and Supplementary Table 3).

### Blood biomarkers

Results for blood biomarkers are shown in Table 2. For cytokines IL-6, IL-7, and IL-15, there were no significant changes between groups. There was a significantly greater increase in insulin in the control group compared to the exercise group (*p* = 0.034), while no significant differences were found for glucose, IGF-1, or IGFBP-1. No significant within-group changes in insulin were observed.

**Table 2:** Changes in blood biomarkers from pre- to post-intervention in the exercise and control groups

	Exercise group (*n*= 11)				Control group (*n*= 6)			
	Pre	Post			Pre	Post		
	Median [IQR]	Median [IQR]	Within-group***p***-value		Median [IQR]	Median [IQR]	Within-group *p*-value	Between groups *p*-value
IL-6 (pg/mL)	8.4 [6.2–12.1]	5.7 [5.1–7.8]	0.965		6.6 [3.2–12.9]	5.1 [2.1–6.8]	1.000	0.910
IL-7 (pg/mL)	6.0 [2.4–23.2]	9.7 [2.5–16.4]	0.721		15.8 [10.6–18.7]	20.4 [9.9–22.9]	0.652†	0.961
IL-15 (pg/mL)	199.0 [112.70–653.4]	154.5 [91.6–441.9]	0.066		406.3 [308.1–800.0]	579.6 [321.4–800.0]	0.940†	0.247
Glucose (mmol/L)	4.7 [4.3–4.9]	4.7 [4.3–4.9]	0.524†		6.5 [6.2–8.1]	7.5 [6.5–8.8]	0.197†	0.171*
Insulin (pmol/L)	83.4 [66.1–112.2]	104.6 [76.5–125.8]	0.838		336.7 [229.0–544.4]	690.9 [311.1–1001.6]	0.272†	**0.034**
IGF-1 (pmol/mL)	72,198.4 [43,679.7–104,222.6]	70,468.2 [47,968.6–79,537.9]	0.646†		59,704.4 [35,020.5–77,060.3]	69,082.0 [66,888.3–80,357.1]	0.223†	0.187*
IGFBP-1 (pmol/mL)	40,624.4 [19,924.8–54,420.9]	36,569.4 [20,353.2–53,511.1]	0.399†		9067.1 [8167.2–12,826.1]	8249.9 [6059.2–14,373.3]	0.834	0.802

*IL* interleukin, *IGF-1* insulin-like growth factor 1, *IGFBP-1* insulin-like growth factor-binding protein-1

*p*-values correspond to between-group comparisons and within-group comparisons of change scores normalised by the number of weeks between pre- and post-assessments. *p*-values were calculated using a Wilcoxon signed-rank test or one-sample *T* test or (marked with †) for within-group comparisons, and a Mann–Whitney *U* test or a Welch’s two-sample *T* test (marked with *) for between-group comparisons. Bold entry indicates a *p*-value <0.05. In the control group, blood samples were collected in a non-fasting state

### Body weight and body composition

In the exercise group, there were no statistically significant changes in body weight and body composition from baseline to post-intervention (Table 3); however, lean mass showed a modest non-significant improvement (MD = 0.80 kg; *p* = 0.093) as did ALM (MD = 0.2 kg; *p* = 0.099).

**Table 3:** Changes in body composition from pre- to post-intervention in the exercise group

	Within-group changes					
	Pre (mean ± SD)	Post (mean ± SD)	MD	95% CI		***p***-value
				Lower	Upper	
% fat mass	36.4 ± 7.0	36.0 ± 6.7	− 0.5	− 1.9	1.0	0.894†
Fat mass (kg)	26.4 ± 9.7	26.2 ± 8.9	− 0.2	− 2.1	1.6	0.785
VAT mass (g)	481.3 ± 325.7	445.6 ± 261.9	− 35.8	− 109.5	38.0	0.424†
Appendicular lean/height^2^ (kg/m^2^)	6.4 ± 1.0	6.6 ± 0.9	0.2	− 0.04	0.4	0.099
Lean mass (Kg)	41.6 ± 4.6	42.4 ± 4.6	0.8	− 0.2	1.8	0.093
% Lean mass	60.6 ± 6.6	61.3 ± 6.2	0.7	− 0.6	2.1	0.859†

*VAT* visceral adipose tissue, *SD* standard deviation, *MD* mean difference, *CI* confidence interval. *p*-values were calculated using a paired *T* test or a Wilcoxon signed-rank test (marked with †)

### Quality of life and fatigue

Patient-reported outcomes are summarised in Table 4 for the exercise group. Quality of life (EORTC QLQ-C30) improved significantly in global health status (+ 16.7 points, 95% CI 1.8–31.5; *p* = 0.031). Although none of the individual functional domains reached statistical significance, improvements were observed across physical (+ 7.9 points), role (+ 3.0 points), emotional (+ 3.8 points), and social functioning (+ 9.1 points).

**Table 4:** Changes in patient-reported outcomes in the exercise group

Within-group changes							
					95% CI		
		Pre (mean ± SD)	Post (mean ± SD)	MD	Lower	Upper	***p***-value
EORTC QLQ-C30							
Function	**Global health**	49.2 ± 24.6	65.9 ± 20.9	16.7	1.8	31.5	**0.031**
	**Physical functioning**	82.4 ± 19.1	90.3 ± 9.6	7.9	− 2.5	18.2	0.121
	**Role functioning**	71.2 ± 28.9	74.2 ± 21.6	3.0	− 13.4	19.5	0.690
	**Emotional function**	62.1 ± 31.9	65.9 ± 21.9	3.8	− 14.0	21.5	0.645
	**Cognitive function**	71.2 ± 19.8	71.2 ± 18.4	0.0	− 18.0	18.0	1.000
	**Social function**	48.5 ± 32.0	57.6 ± 30.1	9.1	− 5.4	23.6	0.192
Symptoms	**Fatigue**	40.4 ± 27.8	37.4 ± 21.2	− 3.0	− 22.5	16.5	0.322†
	**Nausea and vomiting**	9.1 ± 11.5	6.1 ± 8.4	− 3.0	− 11.4	5.4	0.414†
	**Pain**	25.8 ± 31.9	24.2 ± 25.1	− 1.5	− 16.9	13.9	0.785†
	**Dyspnoea**	24.2 ± 30.1	15.1 ± 17.4	− 9.1	− 33.8	15.6	0.432
	**Insomnia**	42.4 ± 33.6	42.4 ± 21.6	0.0	− 26.5	26.5	1.000
	**Appetite loss**	27.2 ± 36.0	18.2 ± 22.9	− 9.1	− 26.7	8.5	0.125†
	**Constipation**	21.2 ± 22.5	18.2 ± 22.9	− 3.0	− 18.7	12.6	0.655†
	**Diarrhoea**	21.2 ± 34.2	15.1 ± 22.9	− 6.1	− 28.0	15.9	0.577†
	**Financial difficulties**	36.4 ± 37.9	36.4 ± 37.9	0.0	0.0	0.0	*
EORTC QLQ-BR23							
Symptoms scales	**Systemic therapy side effects**	51.5 ± 20.2	44.2 ± 19.2	− 7.3	− 22.1	7.4	0.294
	**Hair loss**	54.2 ± 43.4	70.8 ± 33.0	16.6	− 9.1	42.5	0.171
	**Breast symptoms**	18.2 ± 21.8	15.1 ± 15.9	− 3.1	− 14.6	8.6	0.575
	**Arm symptoms**	18.2 ± 17.0	14.4 ± 24.7	− 3.8	− 16.6	9.1	0.395†
Functional scales	**Body image**	55.3 ± 32.3	47.0 ± 34.6	− 8.3	− 31.0	14.3	0.432
	**Future prospective**	33.3 ± 33.3	45.4 ± 27.0	12.1	− 3.0	27.2	0.109†
	**Sexual function**	12.1 ± 16.8	22.7 ± 27.1	10.6	− 5.4	26.6	0.140†
	**Sexual enjoyment**	33.3 ± 47.1	50.0 ± 70.7	16.7	− 1042.2	1075.5	#
FACIT-F							
FACIT-F-TOI		55.5 ± 26.9	63.9 ± 23.0	8.4	− 0.5	17.3	0.063
FACIT-G		67.5 ± 23.6	70.5 ± 18.9	3.0	− 16.2	22.2	0.733
FACIT-Fatigue		91.1 ± 38.4	101.4 ± 29.5	10.3	− 2.5	23.2	0.104

*EORTC QLQ-C30*, European Organisation for Research and Treatment of Cancer Quality of Life Questionnaire-Core 30; *EORTC QLQ-BR23*, European Organisation for Research and Treatment of Cancer Quality of Life Questionnaire-Breast Cancer Module; *FACIT-F*, Functional Assessment of Chronic Illness Therapy-Fatigue; *TOI*, Trial Outcome Index; *G*, general; *SD*, standard deviation; *MD*, meanw difference; *CI*, confidence interval. *p*-values were calculated using a paired *T* test or a Wilcoxon signed-rank test (marked with †). *No statistical analyses conducted because pre- and post-test values ere identical; #Normality test could not be conducted as only two patients completed this score. Bold entry indicates a *p*-value <0.05

Fatigue scores showed a signal toward improvement in the FACIT-F Trial Outcome Index (TOI), with an increase of 8.4 points (*p* = 0.063).

### Response to neoadjuvant chemotherapy

Among patients receiving neoadjuvant chemotherapy (*n* = 7), three of four patients (75%) in the exercise group and all three patients (100%) in the control group achieved a pCR.

## Discussion

This pilot study examined the feasibility and preliminary effects of a 12-week supervised exercise intervention performed during (neo)adjuvant (immuno)chemotherapy on peripheral immune cells, cytokines, metabolic biomarkers, body composition, and patient-reported outcomes in women with breast cancer. Among women who elected to participate, the supervised exercise programme met the predefined feasibility criteria for retention, attendance and adherence rates. Participants in the exercise group showed more pronounced immune changes, particularly within the CD8 + T cell compartment, characterised by decreases in the proportion of naïve and increases in EMRA subsets, together with signals toward decreased proportion of CM CD8 + T cells and increases in the proportion of CD57 and PD-1 expressing T cells compared with controls. No differences were observed in other exhaustion markers in T cells. These results may indicate that exercise during chemotherapy could promote cytotoxic T cell differentiation. Exercise also appeared to preserve metabolic stability, as reflected by a significantly greater increase in insulin in the control group. In addition, patients in the exercise group maintained both fat and lean mass from baseline to end of intervention. Finally, exercisers improved global quality of life and showed a signal toward higher FACIT-F Trial Outcome Index (TOI) score compared with baseline levels.

### Feasibility, safety, and acceptability

High retention (84.6%), attendance (83.6%), and adherence (77.9%) rates indicated that the supervised exercise programme was feasible among women undergoing chemotherapy who chose to participate, with most sessions completed despite treatment-related side effects. Our attendance rate was comparable to the 83% reported in the PACT trial [33]. Similarly, a recent systematic review in breast cancer reported attendance rates of approximately 80% [34], while a meta-analysis in breast cancer reported a pooled exercise adherence of 63% during active treatment [35], although adherence definitions varied considerably across studies. Consistent with these findings, a broader systematic review of exercise interventions during chemotherapy across cancer types reported mean attendance rates of 73.6% and 71.8% for aerobic and resistance exercise sessions, respectively, together with adherence rates of approximately 75% for exercise performed as prescribed [25]

Only one transient, mild hypotensive episode potentially related to exercise occurred. Participant feedback indicated high acceptability, with the intervention perceived as beneficial, manageable, and motivating. However, these feasibility findings should be interpreted in the context of participant self-selection. Women voluntarily enrolled in the exercise intervention and received no financial incentives for participation. Recruitment challenges suggest that the programme may have appealed primarily to a subgroup of women who were particularly motivated to exercise and able to accommodate the demands of participation. Factors such as baseline physical activity levels, proximity to the exercise facility within the study catchment area, access to transport, and sufficient flexibility to attend supervised sessions may have influenced participation. Consequently, while the programme was feasible in this selected sample, its feasibility and scalability may be lower in the broader population of women receiving chemotherapy for breast cancer.

### Immune and metabolic markers

Exercise elicited distinct immunological changes, particularly within CD8 + T cells. Between-group analyses showed greater increases in the proportion of CD8 + EMRA and reductions in CD8 + naïve T cells, with signals toward lower proportion of CD8 + CM, CD8 + naïve CD95 +, and total CD4 + T cells, and increases in CD57 and PD-1 expressing CD8 + T cells in the exercise group compared with controls. In addition, there were within-group increases in CD8 + EMRA, and CD8 + CD57 + T cells, alongside reductions in naïve CD8 + T cells in the exercise group. This suggests an expansion of more antigen-experienced and differentiated CD8 + T cells [36, 37], consistent with previous observations in exercising breast cancer patients during neoadjuvant chemotherapy [23].

On the one hand, the observed increases in CD8 + EMRA and CD8 + CD57 + T cells could suggest a shift toward immunosenescence. Senescent T cells are terminally differentiated with limited proliferation but can secrete high levels of proinflammatory cytokines [38]. On the other hand, EMRA T cells commonly express CD57 and high cytotoxic potential and immediate effector functions despite reduced proliferative ability [39, 40]. In this context, the increased proportion of CD57-expressing CD8 + T cells may instead reflect a functional shift toward a low-proliferative but highly cytotoxic effector state. Further studies are needed to investigate the influence of exercise during chemotherapy by assessing additional senescence-associated markers (e.g. KLRG1, CD27/CD28 loss) or functional assays (e.g. SA-β-gal, SASP).

The proportion of PD-1 expressing CD8 + T cells increased in the exercise group compared with controls. Although PD-1 is a classical marker of exhaustion, its expression can also be transiently upregulated during T cell activation [41]. The absence of changes in other exhaustion markers (CTLA-4, TIM-3, and co-expression patterns) could support the interpretation of activation rather than exhaustion [41]. Nevertheless, we did not observe differences in the proportion of T cells expressing the activation marker HLA-DR. Thus, additional studies are required to evaluate the effects of exercise on activation markers during chemotherapy.

Interestingly, the control group showed a reduction in the proportion of CD4 + T cells co-expressing CTLA-4 and TIM-3. This change could be consistent with a decrease in regulatory T cells (Treg) expressing TIM-3, which has been shown to represent an effector-Treg phenotype with potent suppressive functions on CD4 + helper T cells [42], or a reduction in CD4 + helper T cells expressing TIM-3, which may indicate fewer exhausted CD4 + cells. However, the characterisation of exhaustion in CD4 + T cells is less well established than in CD8 + counterparts [43], and whether this represents a beneficial shift remains unclear and warrants further investigation.

Regarding NK cells, the control group showed a significant increase in CD56bright CD16 + NK cells, a subpopulation considered an intermediate precursor to the cytotoxic CD56dim CD16 + population [44]. This pattern may reflect an expansion of less mature phenotypes, as previously reported in patients with advanced cancer or during chemotherapy [45, 46].

These adaptations could hypothetically be influenced by exercise-induced metabolic and myokine signalling. Muscle-derived metabolites such as lactate and tricarboxylic acid cycle intermediates can enhance the effector profile of CD8 + T cells by downregulating the lymphoid-homing marker CD62L and promoting cytotoxic differentiation [47]. This aligns with the observed reductions in naïve and CM CD8 + cells, which express high levels of CD62L and CCR7, alongside increases EMRA subsets that lack these homing receptors and preferentially circulate in peripheral tissues [37, 40].

The immune-promoting effects of exercise are also partly mediated by the myokines IL-15, IL-7, and IL-6 released from contracting skeletal muscle [7]. IL-15 and IL-7 support T cell homeostasis by promoting thymic output, naïve T cell survival, and maintenance of memory subsets [6, 7]. Moreover, IL-15/IL-7 can induce CD45RA re-expression in CD8 + memory cells [48]. These pathways represent potential mechanisms through which exercise may influence T cell homeostasis, although no significant differences were observed between groups. Similarly, no differences were observed in circulating IL-6 levels. While transient IL-6 release from contracting skeletal muscle acts as a myokine with anti-inflammatory and metabolic benefits and promoting NK cell mobilisation and tumour infiltration [49], chronically elevated IL-6 is a hallmark of tumour-associated inflammation and correlates with advanced disease stage and poorer prognosis in breast cancer [50]. Because IL-6 receptor expression and classical versus trans-signalling pathways were not assessed, the functional relevance of circulating IL-6 in this context remains unclear. Future studies should investigate how chronic exercise influences resting myokine responses and downstream signalling during treatment.

Regarding metabolic biomarkers, differences between groups in insulin were driven largely by an increase in the control group. An insulin rise without parallel glucose change, as observed here, is consistent with compensatory hyperinsulinemia due to emerging peripheral insulin resistance. This aligns with reports of treatment-related glycaemic dysregulation, insulin resistance, and metabolic syndrome driven by adiposity, inflammation, hormonal alterations, and muscle loss [51, 52]. These preliminary patterns suggest a potential protective effect of exercise against chemotherapy-related insulin dysregulation, consistent with its role in enhancing skeletal muscle insulin sensitivity and GLUT4-mediated glucose uptake [53] and with meta-analyses showing improvements in fasting glucose and insulin with combined aerobic and resistance training during and after chemotherapy [54].

Nevertheless, given the study’s design, these immune and metabolic findings must be interpreted cautiously. The mechanistic explanations are exploratory, and alternative interpretations, including treatment effects, differences in fasting status between groups for metabolic markers, or individual variability, cannot be excluded. Larger, mechanistically focused studies are needed to confirm these observations.

### Body composition

Patients in the exercise group maintained stable body weight, fat mass, and lean mass during chemotherapy. This may imply a protective effect against the typical chemotherapy-induced increases in fat mass [52, 55] and loss of lean mass [55, 56]. Such preservation of lean tissue and attenuation of fat gain may carry clinical relevance, as higher lean mass and lower adiposity have been associated with better pCR to neoadjuvant chemotherapy [57], whereas post-diagnosis increases in body mass index, body fat, or waist circumference predict poorer breast cancer–specific and overall survival [58]. These results align with evidence showing that exercise mitigates adverse body composition changes during chemotherapy [55].

### Patient-reported outcomes

Cancer and its treatments commonly impair QoL and increase fatigue [59, 60]. We observed significant improvements in QoL and a signal toward reduced fatigue, consistent with prior work demonstrating that exercise enhances QoL [61] and reduces cancer-related fatigue [62]. Although no individual functional domain reached statistical significance, modest improvements were observed across several domains, suggesting that the improvement in global health status may reflect small cumulative benefits across multiple areas. Given the supervised group-based nature of the intervention, we cannot exclude that social interaction may also have contributed to these improvements.

### Treatment response

The clinical relevance of these effects remains to be determined. In our study, all patients in the control group and all but one in the exercise group achieved a pCR, the latter being a patient with inflammatory breast cancer, a subtype typically associated with lower pCR rates [63]. Given the small sample size and uniformly high pCR rate, no conclusions can be drawn regarding group differences, but future studies should assess the relationship between exercise-induced immune modulation and response to neoadjuvant chemotherapy.

### Limitations and strengths

The main limitation of this study is the limited generalisability of the findings. Participants self-selected into the exercise intervention, introducing the potential for selection bias. As discussed above, the observed feasibility may reflect the characteristics of participants who were able and willing to engage in a supervised exercise programme during chemotherapy and therefore may not extend to the wider breast cancer population. Additional limitations include the small sample size, the inclusion of a convenience control group, unequal group sizes, and baseline differences in menopausal status between groups, which limit between-group comparability and preclude causal inference. Variability in chemotherapy regimens, differences in the timing of blood sampling relative to chemotherapy infusions, and the collection of non-fasted blood samples in the control group due to logistical constraints may also have influenced circulating immune and metabolic markers, although statistical normalisation for sampling intervals was applied to partly account for these differences. Lastly, functional immune assays and analyses of tumour immune cell infiltration were not performed, limiting the ability to determine whether observed phenotypic changes translate into meaningful alterations in immune cell function and enhanced tumour infiltration. Despite these limitations, the study’s strengths include the comprehensive phenotyping of peripheral immune cells and the concurrent assessment of cytokines and metabolic biomarkers, body composition, and patient-reported outcomes.

### Conclusions

In conclusion, our pilot study of a 12-week supervised combined aerobic- and resistance exercise programme performed during (neo)adjuvant chemotherapy was feasible, safe, and acceptable in a self-selected group of women with breast cancer who chose to participate. Exploratory analyses indicated potential immunological changes, particularly within the CD8 + T cell compartment, characterised by an expansion of terminally differentiated antigen-experienced subsets in exercisers compared with controls, while controls showed a signal toward a greater expansion in CD56 bright CD16 + NK cells. Moreover, exercise participants also showed a different insulin trajectory compared with controls. Furthermore, despite ongoing treatment, patients preserved body weight, fat mass and lean mass, improved global quality of life, and reduced fatigue. Given the exploratory nature of this pilot study, the small sample size, and the non-randomised design, these findings should be interpreted with caution. Larger studies are needed to confirm these preliminary results and to determine if exercise-induced immune and myokine changes translate into improved therapeutic response and long-term outcomes.

## Supplementary Information

Below is the link to the electronic supplementary material.

ESM 1 (DOCX 1.18 MB)

## Data Availability

The datasets generated during and/or analysed during the current study are available from the corresponding author on reasonable request.
