# Supplementary material for: Exploring the feasibility of exercise training during (neo)adjuvant chemotherapy in women with early-stage breast cancer: a pilot study examining immune and metabolic outcomes
Source: Support Care Cancer. 2026 Sep 24;34(10):1013. doi: 10.1007/s00520-026-11179-8 (PMC13612707; doi:10.1007/s00520-026-11179-8)
Supplement: Supplementary file 1 [file 520_2026_11179_MOESM1_ESM.docx]

## **6. Supplementary material**

**Figure 1. Gating strategy**

**D**

(A) Sequential gating of lymphocytes, single and live cells, followed by identification of CD3+CD4+ and CD3+CD8+ T-cell populations.
(B) Gating strategy for CD8+ T-cell subsets based on CD45RA and CCR7 expression (naïve, CM, EM, EMRA) and further characterization of CD8+ T cells expressing CD95, HLA-DR, CD57, PD-1, CTLA-4 and TIM-3.
(C) Gating strategy for CD4+ T-cell subsets based on CD45RA and CCR7 expression (naïve, CM, EM, EMRA) and further characterization of CD4+ T cells expressing CD95, HLA-DR, CD57, PD-1, CTLA-4 and TIM-3.
(D) Gating of NK (CD3⁻CD56+) and NKT-like (CD3+CD56+) cells and NK subsets based on CD56 and CD16 expression (CD56bright CD16⁻, CD56bright CD16+, CD56dim CD16+, CD56dim CD16⁻).

**Figure 2. Changes in CD4 T cell subsets from pre- to post-intervention in the exercise and control groups**

*****

**# †**

**% CD4+ of CD3+**

**A**

Changes in peripheral CD4 T cell subsets from baseline (pre) to end of intervention (post) in the exercise (grey, n = 11) and control (pink, n = 6) groups. Data are shown as individual values and group means. Ex: exercise; Con: control.

**#**

(A) % CD4+ of CD3+ cells; (B) % CD4+ Naïve; (C) % CD4+ CM; (D) % CD4+ EM; (E) % CD4+ EMRA; (F) % CD4+ HLA-DR+; (G) % CD4+ CD57+; (H) CD4+ PD-1+.

Signals toward significance (p < 0.10) are indicated by #, and statistically significant differences (p < 0.05) by *.

*p*-values correspond to between-group comparisons and within-group comparisons of change scores normalised by the number of weeks between pre- and post-assessments.

Abbreviations: CM, central memory; EM, effector memory; EMRA, effector memory re-expressing CD45RA.

**Figure 3. Changes in NK cells from pre- to post-intervention in exercise and control groups**

Changes in peripheral immune cell subsets from baseline (pre) to end of intervention (post) in the exercise (grey, n = 11) and control (pink, n = 6) groups. Ex: exercise; Con: control. Data are shown as individual values and group means.

(A) % total NKT-like cells of lymphocytes; (B) % total NK cells of lymphocytes; (C) % CD56bright CD16+ NK cells; (D) % CD56bright CD16- NK cells; (E) % CD56dim CD16+ NK cells; (F) % CD56dim CD16- NK cells.

Signals toward significance (p < 0.10) are indicated by #, and statistically significant differences (p < 0.05) by *. *p*-values correspond to between-group comparisons and within-group comparisons of change scores normalised by the number of weeks between pre- and post-assessments.

**Table 1. Volume of resistance training**

| **Exercise** | **Load Pre (kg) Mean (SD)** | **Load Post (kg) Mean (SD)** | **Δ Load (kg) Mean (SD)** | **% change** | **Volume Pre (kg)**  **Mean (SD)** | **Volume Post (kg)**  **Mean (SD)** | **Δ Volume (kg)**  **Mean (SD)** | **% change** |
| --- | --- | --- | --- | --- | --- | --- | --- | --- |
| **Leg Press (n=9)** | 42.9  (11.6) | 52.5  (11.1) | 9.6  (6.8) | 27.4  (26.2) | 857.3  (232.5) | 1680.5  (354.8) | 823.3  (210.4) | 103.8  (41.9) |
| **Squats with dumbbells (n=2)** | 8 | 11 | 3 | 43.3 | 160 | 352 | 192 | 129.3 |
| **Leg curl** | 19.7  (4.0) | 24.0  (2.6) | 4.3  (3.9) | 27.4  (35.9) | 394.7  (80.6) | 767.8  (82.7) | 373.0  (92.2) | 103.9  (57.5) |
| **Leg extension** | 18.4  (5.1) | 22.1  (4.8) | 3.8  (3.26) | 22.9  (21.1) | 367.8  (102.6) | 708.9  (153.9) | 341.1  (96.7) | 96.6  (33.7) |
| **Calf raises with dumbbells** | 5.5  (3.5) | 7.6  (4.1) | 2.1  (3.4) | 50.8  (74.2) | 112.7  (67.1) | 247.3  (124.0) | 134.5  (96.3) | 177.6  (198.0) |
| **Chest Press** | 10.2  (4.4) | 13.6  (3.9) | 3.4  (3.6) | 60.1  (90.1) | 204.0  (88.2) | 434.9  (123.5) | 230.9  (96.5) | 156.1  (144.2) |
| **Seated row** | 18.8  (5.7) | 25.6  (6.1) | 6.7  (2.7) | 39.7  (23.1) | 377.1  (114.2) | 818.9  (196.3) | 441.8  (105.7) | 123.5  (37.0) |
| **Lat Pulldown** | 18.5  (5.8) | 23.0  (4.8) | 4.5  (5.1) | 31.3  (35.1) | 370.0  (115.9) | 736.4  (155.1) | 366.5  (133.6) | 110.0  (56.1) |
| **Shoulder press with dumbbells** | 5.9  (2.6) | 7.7  (2.6) | 1.8  (1.0) | 42.4  (54.1) | 118.2  (52.5) | 245.8  (84.1) | 127.6  (41.0) | 127.9  (86.5) |
| **Triceps extension** | 7.5  (1.9) | 8.2  (1.9) | 0.7  (1.3) | 10.5  (20.0) | 149.7  (38.1) | 261.0  (62.4) | 111.3  (40.7) | 76.9  (32.0) |
| **Biceps curl** | 6.5  (2.5) | 7.9  (2.8) | 1.4  (1.4) | 31.8  (57.8) | 130.9  (50.9) | 253.1  (91.0) | 122.2  (53.6) | 110.9  (92.5) |

Volume Pre (kg) was calculated as kg × reps × sets. Δ values represent post-pre differences. Percentage change was calculated as (Post-Pre)/Pre) × 100. For baseline values of zero, the smallest nonzero baseline observed for that exercise was used as a correction.

**Table 2. Acceptability of the program**

| **QUESTIONS** | **Median rating**  **(1=not at all; 7= very much) (IQR)** |
| --- | --- |
| **1.How much of a burden was it for you to complete each of the following aspects of the study?** |  |
| a) The questionnaires | 1 (1-1.5) |
| b) the exercise program | 1 (1-2) |
|  | |
| **2. How do you feel about having taken part in the exercise research study?** |  |
| (a) rewarding | 7 (7-7) |
| (b) a waste of my time | 1 (1-1) |
| (c) useful for research helping others | 7 (7-7) |
| (d) useful for me personally | 7 (7-7) |
| (e) something that I would recommend to other people with the same cancer as me | 7 (7-7) |
|  | |
| **3.How much of a barrier was each of the following factors for you in trying to do the exercise program?** |  |
| (a) feeling tired or fatigued | 3 (3-4.5) |
| (b) symptoms and side effects of treatments | 4 (3-4) |
| (c) other medical/health problems | 2 (1-3) |
| (d) too busy and had limited time | 3 (1.5-3.5) |
| (e) pain or soreness | 2 (1-2.5) |
| (f) feeling sick/not feeling well | 4 (2.5-4.5) |
| (g) medical appointments | 2 (1-2.5) |
| (h) lack of motivation | 1 (1.-2.5) |
|  | |
| **Were there any other barriers that you experienced?** | |
| - ‘Having a PICC line’ - ‘I am not driving so I had to organise a lift to each session’ - ‘The time slot, as traffic is very bad on freeway at that time of day’ - ‘Transport’ - ‘Location-30-minute drive. Fortunate that family or friends drove/joined me when I was too fatigued’ - ‘Having cash on hand for parking’ - ‘It was a bit far, but the people and benefits were amazing. Loved my coach and everyone I exercised with’ | |
| **Is there anything else you would like to tell us?**  On this final page, please feel free to make any comments concerning your medical condition, the questionnaire itself, the program, or anything else you think may be helpful to us or that you want to share. All comments are welcome. | |
| - *"Exercise while having chemo really helped with the side effects, and if I were ever to give advice to anyone with breast cancer, it would be exercise. It was also nice to have Cristina with me throughout my chemo journey to keep me motivated to move."* - *"I loved this program-it helped me so much both mentally and physically. I wish it had lasted throughout my entire treatment. Cristina was amazing: so reassuring, helpful, and encouraging. I would recommend it to anyone going through chemo; it helped me immensely."* - *"I would highly recommend the FASTEX trial to other patients. The program kept me active, accountable, and motivated to focus on my health. It improved my overall physical and mental wellbeing. I experienced very few side effects during treatment. Cristina did a great job ensuring the exercise was safe and tailored to each participant's needs, and she always encouraged us to do our best and stay positive. Thank you!"* - *"Cristina provided a welcoming and supportive environment, which-along with the exercise-made participating a pleasure and improved my overall wellbeing."* - *"I found the exercise program to be quite rewarding and helpful. As a woman who is not very fit, I felt the program was exactly what I needed. Thank you, Cristina, for your guidance."* - *"All cancer patients should be encouraged to exercise or join the program, as it was mentally and physically beneficial."* - *"The program kept me accountable to being active during treatment. It provided structure and routine. Even on the days when I struggled to get to class and had little energy, I always left feeling better than when I arrived. I found the connections I made through the program to be valuable to my wellbeing."* - *"This exercise program got me through some of the hardest days of my life. Had it not been for this program, I feel my mental health would have been in a terrible state. I am so grateful I got to participate."* - *"Cristina was amazing-so friendly and helpful-and I really enjoyed the program. I hope she gains valuable insights from her clients and completes her PhD. Thank you :)"* - *"Great program and motivator."* - *"One of the best programs. It should be made compulsory for every cancer patient."* | |

Responses were rated on a 7-point Likert scale ranging from 1 (“not at all”) to 7 (“very much”), with higher scores indicating greater agreement or endorsement of the item.

**Table 3. Changes in T cells expressing exhaustion markers from pre- to post-intervention in exercise and control groups**

|  | Exercise | | | Control | | |  |
| --- | --- | --- | --- | --- | --- | --- | --- |
|  | **Pre** | **Post** |  | **Pre** | **Pre** |  |  |
|  | Mean (SD) | Mean (SD) | Within group  *p*-value | Mean (SD) | Mean (SD) | Within group  *p*-value | Between groups  *p*-value |
| CD8+CTLA4+ | 2.8 (2.7) | 1.7(0.8) | 0.286 † | 3.5 (1.7) | 2.6 (1.5) | 0.200 | 0.357* |
| CD8+PD1+/CTLA4+ | 0.4 (0.2) | 0.3 (0.2) | 0.662 | 0.5 (0.2) | 0.3 (0.1) | 0.200 | 0.231 |
| CD8+TIM3+ | 13.6(5.6) | 13.0(4.0) | 0.658 | 21.1 (3.8) | 20.5 (6.6) | 0.675 † | 0.786* |
| CD8+TIM3+/CTLA4+ | 0.6 (0.6) | 0.4 (0.2) | 0.126 † | 0.4 (0.5) | 0.3 (0.2) | 0.805 | 0.245* |
| CD4+CTLA4+ | 4.1 (2.8) | 4.0 (3.5) | 0.973 | 4.8 (1.0) | 3.5 (1.9) | 0.208 † | 0.175* |
| CD4+PD1+/CTLA4+ | 0.6 (0.3) | 0.6 (0.3) | 0.553 | 0.9 (0.3) | 0.6 (0.3) | 0.138 | 0.165 |
| CD4+TIM3+ | 4.7 (3.0) | 4.6 (1.2) | 0.847 | 5.2 (1.8) | 4.7 (2.0) | 0.812 | 0.865 |
| CD4+TIM3+/CTLA4+ | 0.6 (0.4) | 0.5 (0.3) | 0.355 | 0.5 (0.1) | 0.3 (0.1) | 0.019 | 0.052 |

Values are expressed as mean (SD). *p*-values correspond to between-group comparisons and within-group comparisons of change scores normalised by the number of weeks between pre- and post-assessments. *p*-values were calculated using one-sample T test or a Wilcoxon signed-rank test (marked with †) for within-group comparisons, and Welch’s two-sample T test or a Mann-Whitney U test (marked with *) for between-group comparisons.

Abbreviations: SD, standard deviation; PD-1, programmed cell death protein 1; CTLA-4, cytotoxic T-lymphocyte-associated antigen 4; TIM-3, T-cell immunoglobulin and mucin domain-3.
